# The *Cannabis sativa* Versus *Cannabis indica* Debate: An Interview with Ethan Russo, MD

**DOI:** 10.1089/can.2015.29003.ebr

**Published:** 2016-01-01

**Authors:** Daniele Piomelli, Ethan B. Russo

**Affiliations:** ^1^ Department of Anatomy and Neurobiology, University of California, Irvine, Irvine, California.; ^2^ Department of Drug Discovery and Development, Istituto Italiano di Tecnologia, Genoa, Italy.; ^3^ PHYTECS, Los Angeles, California.

## Abstract

Dr. Ethan Russo, MD, is a board-certified neurologist, psychopharmacology researcher, and Medical Director of PHYTECS, a biotechnology company researching and developing innovative approaches targeting the human endocannabinoid system. Previously, from 2003 to 2014, he served as Senior Medical Advisor and study physician to GW Pharmaceuticals for three Phase III clinical trials of Sativex^®^ for alleviation of cancer pain unresponsive to optimized opioid treatment and studies of Epidiolex^®^ for intractable epilepsy. He has held faculty appointments in Pharmaceutical Sciences at the University of Montana, in Medicine at the University of Washington, and as visiting Professor, Chinese Academy of Sciences. He is a past President of the International Cannabinoid Research Society and former Chairman of the International Association for Cannabinoid Medicines. He serves on the Scientific Advisory Board for the American Botanical Council. He is the author of numerous books, book chapters, and articles on Cannabis, ethnobotany, and herbal medicine. His research interests have included correlations of historical uses of Cannabis with modern pharmacological mechanisms, phytopharmaceutical treatment of migraine and chronic pain, and phytocannabinoid/terpenoid/serotonergic/vanilloid interactions.

***Cannabis and Cannabinoid Research*** **(Dr. Daniele Piomelli:** ***CCR*): I would like to start with a few questions that should not be too contentious. First off, what is the geographic origin of the Cannabis plant?**

**Dr. Russo:** *Cannabis* originated in Central Asia and perhaps the Himalayan foothills. There are converging lines of evidence, including a center of biological diversity there, and biochemical data that support this. There is no trace of its presence in the Western Hemisphere before the 16th century.

***CCR*: What chemical(s) are the major contributors to the psychoactive effects of Cannabis? Δ^9^-tetrahydrocannabinol, cannabidiol, or others?**

**Dr. Russo:** Δ^9^-tetrahydrocannabinol is, of course, the pre-eminent psychoactive component of Cannabis. Δ^8^-tetrahydrocannabinol, a more heat-stable component, is probably slightly less psychoactive, but is present only in trace amounts or as an artifact of laboratory analysis. Cannabinol is the nonenzymatic oxidative breakdown product of tetrahydrocannabinol (THC), seen in aged Cannabis, and is about 25% of the potency of THC. Tetrahydrocannabivarin (THCV) is a neutral antagonist at CB_1_ at low doses, but an agonist at high doses, and is certainly psychoactive, but rarely seen in high titer in commonly available Cannabis strains. Finally, although cannabidiol (CBD) is nonintoxicating, it certainly has antianxiety, antipsychotic, and even antidepressant effects, so properly they must be considered psychoactive with these qualifications.

***CCR*: What about other medicinal properties of the plant? For example, the local anti-inflammatory actions extolled by some ancient writers?**

**Dr. Russo:** CBD is a versatile anti-inflammatory analgesic through numerous distinct mechanisms, and various other minor cannabinoids and terpenoids in Cannabis certainly may contribute notably to the therapeutic profile of Cannabis. Numerous basic science and even clinical trial data support the concept of herbal synergy in Cannabis beyond the effects of single components.^1–3^ We are only seeing the very beginnings of the therapeutic potential of this plant!

***CCR*: Can you explain what is meant by entourage effect as it pertains to Cannabis?**

**Dr. Russo:** This concept was first espoused by Drs. Mechoulam and Ben-Shabat more than 15 years ago to explain how certain components of the endocannabinoid system boost the therapeutic effects of its main players, anandamide and 2-arachidonylglycerol.^4,5^ Thus, it is akin to a symphony, in which many musicians support and harmonize the melody provided by the soloists. The same analogy fits well the synergistic phenomena observed in Cannabis, whose various components boost and compliment those of its better known ones, THC and CBD.

***CCR*: People have been selecting Cannabis strains for quite some time now. One would expect human selection to have substantial effects on Cannabis' psychoactive and medicinal properties. Is this true?**

**Dr. Russo:** Absolutely! While there have always been very potent Cannabis strains to be found, they are certainly more commonly available today due to selective breeding and culture techniques that produce *ganja*, or *sinsemilla*, that is, unfertilized female flowers. The plant puts all its energy into production of cannabinoids and Cannabis terpenoids instead of producing seeds. Unfortunately, until recently, almost all the effort in breeding has been toward higher potency THC strains rather than on the safer and arguably much more therapeutically versatile mixed or CBD predominant strains. Selective breeding for medicinal efficacy is a relatively new phenomenon that is now accelerating.

***CCR*: Now, moving onto something more controversial. Here is a statement one can find on the Web: “It is widely accepted that marijuana has two different species:** ***Cannabis indica*** **and** ***Cannabis sativa*.” This was of course also the opinion of the great 18th century naturalist, Jean-Baptiste Lamarck, but would academic botanists today agree with this statement?**

**Dr. Russo:** Botanical taxonomists never agree on anything for very long! To paraphrase and expropriate an old Yiddish expression: 12 botanical taxonomists, 25 different opinions. Many classical botanists would argue for Cannabis as one polymorphic species based on the ability of all its types to interbreed. However, if this were true, hundreds of neotropical gesneriads (Gesneriaceae, members of the African violet family) would all be one species since they readily hybridize and produce fertile offspring. It is clear that there are many chemotypes of Cannabis: THC predominant, CBD predominant, and mixed types. This is a good basic classification, but it has also been possible to selectively breed for other chemotypes expressing high titers of THCV, cannabidivarin, cannabichromene, and even ones producing 100% of its cannabinoids as cannabigerol, or others with no cannabinoids at all. The debate continues. Some espouse Cannabis as a single species, while others describe up to four: *Cannabis sativa*, *Cannabis indica*, *Cannabis ruderalis*, and *Cannabis afghanica* (or *kafiristanica*).^6,7^

***CCR*: Some users describe the psychoactive effects of** ***Cannabis indica*** **and** ***sativa*** **as being distinctive, even opposite. But are they really? Beyond self-reports from users, is there any hard evidence for pharmacologically different species of Cannabis?**

**Dr. Russo:** There are biochemically distinct strains of Cannabis, but the *sativa*/*indica* distinction as commonly applied in the lay literature is total nonsense and an exercise in futility. One cannot in any way currently guess the biochemical content of a given Cannabis plant based on its height, branching, or leaf morphology. The degree of interbreeding/hybridization is such that only a biochemical assay tells a potential consumer or scientist what is really in the plant. It is essential that future commerce allows complete and accurate cannabinoid and terpenoid profiles to be available.

***CCR*:** ***Sativa*** **is often described as being uplifting and energetic, whereas** ***indica*** **as being relaxing and calming. Can you speculate on what could be the basis for these perceived differences?**

**Dr. Russo:** We would all prefer simple nostrums to explain complex systems, but this is futile and even potentially dangerous in the context of a psychoactive drug such as Cannabis. Once again, it is necessary to quantify the biochemical components of a given Cannabis strain and correlate these with the observed effects in real patients. Beyond the increasing number of CBD predominant strains in recent years, almost all Cannabis on the market has been from high-THC strains. The differences in observed effects in Cannabis are then due to their terpenoid content, which is rarely assayed, let alone reported to potential consumers. The sedation of the so-called *indica* strains is falsely attributed to CBD content when, in fact, CBD is stimulating in low and moderate doses! Rather, sedation in most common Cannabis strains is attributable to their myrcene content, a monoterpene with a strongly sedative couch-lock effect that resembles a narcotic. In contrast, a high limonene content (common to citrus peels) will be uplifting on mood, while the presence of the relatively rare terpene in Cannabis, alpha-pinene, can effectively reduce or eliminate the short-term memory impairment classically induced by THC.^2,8^

***CCR*: How do you think one could address the** ***sativa*/*indica*** **dichotomy in a scientifically sound manner?**

**Dr. Russo:** Since the taxonomists cannot agree, I would strongly encourage the scientific community, the press, and the public to abandon the *sativa*/*indica* nomenclature and rather insist that accurate biochemical assays on cannabinoid and terpenoid profiles be available for Cannabis in both the medical and recreational markets. Scientific accuracy and the public health demand no less than this.

***CCR*:** Thank you, Dr. Russo. We all appreciate your insight into this controversial, complex, and very important topic.

## Abbreviations Used

CBD: cannabidiol
THC: tetrahydrocannabinol
THCV: tetrahydrocannabivarin

## References

[1] Johnson JR, Burnell-Nugent M, Lossignol D, et al. Multicenter, double-blind, randomized, placebo-controlled, parallel-group study of the efficacy, safety, and tolerability of THC:CBD extract and THC extract in patients with intractable cancer-related pain. J Pain Symptom Manage. 2010;39:167–179 19896326 10.1016/j.jpainsymman.2009.06.008

[2] Russo EB Taming THC: potential cannabis synergy and phytocannabinoid-terpenoid entourage effects. Br J Pharmacol. 2011;163:1344–1364 21749363 10.1111/j.1476-5381.2011.01238.x PMC3165946

[3] Russo EB, Guy GW A tale of two cannabinoids: the therapeutic rationale for combining tetrahydrocannabinol and cannabidiol. Medical Hypotheses. 2006;66:234–246 16209908 10.1016/j.mehy.2005.08.026

[4] Ben-Shabat S, Fride E, Sheskin T, et al. An entourage effect: inactive endogenous fatty acid glycerol esters enhance 2-arachidonoyl-glycerol cannabinoid activity. Eur J Pharmacol. 1998;353:23–31 9721036 10.1016/s0014-2999(98)00392-6

[5] Mechoulam R, Ben-Shabat S From gan-zi-gun-nu to anandamide and 2-arachidonoylglycerol: the ongoing story of cannabis. Nat Prod Rep. 1999:16:131–143 10331283 10.1039/a703973e

[6] Clarke RC, Merlin MD Cannabis: evolution and ethnobotany. University of California Press: Berkeley, CA, 2013

[7] Small E Evolution and classification of *Cannabis sativa* (marijuana, hemp) in relation to human utilization. Bot Rev. 2015;81:189–294

[8] Komori T, Fujiwara R, Tanida M, et al. Effects of citrus fragrance on immune function and depressive states. Neuroimmunomodulation. 1995;2:174–180 8646568 10.1159/000096889

